# Supplementary material for: Are carbon emissions trading and green financial instruments synergistic? -Comprehensive quantitative research based on content analysis
Source: PLoS One. 2024 Mar 7;19(3):e0298601. doi: 10.1371/journal.pone.0298601 (PMC10919601; doi:10.1371/journal.pone.0298601)
Supplement: S1 Data — (ZIP) [file pone.0298601.s001.zip › Data/地级市Z_F工作报告.zip!/║╙─╧/║╫▒┌╩╨/└┤╫╘╣1⁄2╓┌║┼í╛┬φ┐╦╩2╛▌═°í┐_2007.docx]

各位代表：

　　现在，我代表市人民政府，向大会作政府工作报告，请予审议，并请各位政协委员提出意见。

　　一、2006年工作回顾

　　2006年是我市全面实施“十一五”规划的重要一年。一年来，市政府在省委、省政府和市委的正确领导下，团结带领全市人民，全面落实科学发展观，努力构建和谐社会，开拓创新，扎实工作，圆满完成了市八届人大三次会议确定的各项任务，被省政府评为完成责任目标优秀单位，实现了“十一五”发展的良好开局。

　　经济发展水平整体提升。初步核算，全市生产总值突破200亿元，比上年增长16.8%，增速首次居全省第一位；人均生产总值突破15000元。财政一般预算收入突破10亿元，增长31%，居全省第二位；支出20.7亿元，增长13.4%。全社会固定资产投资突破100亿元，增长46%，其中城镇投资96.9亿元，增长47.2%，居全省第二位。粮食总产突破100万吨，单产达到416公斤，连续3年创历史最好水平。畜牧业产值、加工业产值分别达到40.2亿元和51亿元，畜牧业占农业总产值的比重超过60%。规模以上工业增加值突破100亿元，增长25.3%；实现利润17.2亿元，增长123.7%，两项指标增速均居全省第三位。煤电、食品加工、高新技术、纺织服装、新型建材、汽车及零部件、金属镁、煤化工产业实现增加值75亿元，占规模以上工业增加值的70.2%，优势特色产业集群初见规模与形象，整体活力明显增强。第三产业发展加快，增加值增长11.2%，被列为省服务业体制改革和创新试点市。本地金融机构各项存款余额增长21.8%，居全省第一位；各项贷款余额增长15.6%；吸引异地金融机构贷款余额28亿元，增长124.3%，是近年来吸引市外信贷投放最多的一年。城镇居民人均可支配收入、农民人均纯收入分别突破9000元和4000元，其中城镇居民人均可支配收入水平在全省前移3个位次；实际增长14.3%和15.6%，分别居全省第2位和第6位。社会消费品零售总额46.6亿元，增长15.3%。

　　招商引资和大项目建设取得新进展。全年引进市外直接投资75.8亿元，实际利用境外直接投资3970万美元。281个新落地项目中，超亿元项目26个，投资规模平均达到4153万元。工业集中区新入驻企业73家，总投资33.8亿元。大项目建设再创佳绩，66个重点项目建成34个、在建28个，完成投资60.1亿元，比上年增加24.1亿元。电厂三期完成投资13.3亿元，累计完成总投资的47.5%。惠及10万群众的采煤沉陷综合治理项目完成投资3.1亿元，累计完成总投资的57.8%。热电联供项目完成投资4.57亿元，累计完成总投资的91.2%，1号机组并网发电。新老区快速通道试通车。盘石头水库移民工作经过艰苦努力取得重要进展。加大协调和服务力度，60万吨甲醇、鹤辉高速公路、长鹤泰铁路、京郑铁路客运专线、京珠高速公路拓宽、南水北调中线工程等一批对全市发展具有重要支撑作用的大项目，均取得积极进展。招商引资和大项目的快速推进，实现了我市经济基础由薄弱到比较扎实、产业结构由单一到多元化的重要转变，为今后更好更快发展奠定了较为坚实的基础。

　　循环经济发展成效明显。把循环经济发展与节约型社会建设、环境保护等紧密结合起来，下大力气加以推进，在全国发展循环经济电视电话会议上作了典型发言，吴邦国委员长、省委省政府都对我市循环经济发展给予了高度评价。循环经济试点实施方案通过国家评审并组织实施。总投资34.3亿元的28个循环经济重点项目全部开工建设，完成投资24.6亿元。鹤煤集团煤层气发电、矿井水综合利用、煤矸石烧结砖、粉煤灰制品等项

　　2月6日，市长李培育在市八届人大四次会议上作《政府工作报告》。袁国强摄

　　目进展顺利。秸秆发电项目完成总投资的93%。电厂二期脱硫项目完成总投资的77%。电厂一期脱硫、同力水泥余热发电等项目开工建设。4个循环经济示范区和16个示范企业建设全面展开。对40户重点能耗企业实施节能目标管理，对7家企业进行强制性清洁生产审核，节能降耗取得初步成效。建筑节能工作扎实推进，建设花苑1号、2号楼成为全省首家达到65%节能标准的居住建筑，我市被确定为全国建筑节能试点市。关闭黏土砖瓦窑场94家，一年完成省政府下达的两年“禁实”目标任务。增加新型墙材产能5.4亿标块。环境保护和生态建设全面加强。地表水省控责任目标断面水质平均达标率实现100%，同比提高24.6个百分点，创历年最好水平；城市饮用水源地水质达标率保持100%；环境空气质量优良天数达到335天，比上年增加34天，进入省先进行列。涉及19家企业的卫河流域水污染综合整治年度任务圆满完成。工业污染源排放达标率和污染防治设施正常运转率均超过95%。老区居民关注的化工企业搬迁整治工作开始启动。浚县、淇县污水处理厂试运行。市垃圾处理场一期工程基本建成。城市环境综合整治定量考核工作居全省第8位，比上年前移6个位次。完成造林12万亩，平原绿化新建和完善林网37万亩，实现了全市平原绿化整体高级达标。

　　城乡一体化步伐加快。城镇化率达到44.18%，比全省平均水平高11.68个百分点。新区建设形成新高潮。完成城建投资15亿元，比上年增长38%；新开工建筑面积突破200万平方米，建成区面积新增5平方公里，达到22平方公里；城市绿化覆盖率达到40.4%。新区增加供热面积110万平方米。市体育馆、湘江小学、特殊教育学校开工建设。淇河公园建设全面铺开。老干部活动中心建成投入使用。大赉店等城中村改造取得积极进展，南部两个村民安置小区一期工程开工建设。老区改造建设扎实推进，管理力度继续加大，城市功能和人居环境得到新的改善，潜能得到释放，活力进一步增强。成功承办了中国城市科学研究会中小城市分会第17次年会，荣获中国人居环境范例奖。两县县城改造建设与扩张加快，小城镇面貌得到改观，经济实力和人口吸纳力得到增强。新农村建设全面扎实推进，农民生产生活条件明显改善。龙头企业的带动能力进一步增强，农民专业合作组织和行业协会新增110家，农业产业化水平明显提高。新增食品加工能力60万吨，年加工能力达到276万吨。71个新农村建设示范村规划全部完成，村容村貌明显改观。农村中小学现代远程教育、乡镇卫生院改造、道路建设等工作推进快、质量好，走在了全省前列。撤并调整中小学120所，进一步优化了农村教育资源配置。建立了农村中小学校舍维修改造长效机制。提高了农村中小学公用经费标准。新改造乡镇卫生院9所，成为全省唯一全部完成乡镇卫生院改造的省辖市。建成投用100所标准化村卫生室，7所乡镇文化站和30个示范性村文化大院，8处乡镇体育健身广场、10个农民体育健身工程。完成“村村通”油(水泥)路560公里，通达行政村503个，提前一年实现全市所有行政村“村村通”油(水泥)路；建设县乡公路46公里，提前一年实现所有乡镇拥有二级以上公路出口；建成25个乡镇客运站，提前一年完成省定目标；建成856个招呼站，提前4年完成市“十一五”目标。行政村光缆覆盖率达到100%；110个20户以上的自然村实现了通广播电视，提前4年在全国率先完成“十一五”目标。“万村千乡”市场工程试点工作新建农村连锁超市329个，全市半数以上的行政村有了连锁超市，方便了农民生活。创新、出台了户籍、土地、就业、社会保障等7个方面9项政策措施，为城乡一体化提供了更好的体制机制和政策环境。

　　和谐社会建设有效推进。投入5.7亿元，圆满完成了省委、省政府承诺实事中涉及我市的年度任务和市委、市政府承诺的十件实事。城镇就业再就业和农村富余劳动力转移工作再创佳绩，下岗失业人员再就业培训1.4万人，实现城镇就业4.2万人、下岗失业人员再就业2万人，完成省定目标比例均居各省辖市前列；对2.4万名农村劳动力进行技能培训，劳务输出20.7万人；在全省率先提出并解决了全市704户“零就业家庭”就业问题；被确定为全国统筹城乡就业试点市。社会保险覆盖面进一步扩大，企业养老、失业、医疗、工伤保险参保人数分别达到9.9万人、15.3万人、16.6万人和9.5万人。城乡社会救助体系进一步完善，慈善事业健康发展，7.9万名城乡困难群众纳入低保，实现了动态管理下的应保尽保；市区、两县低保标准分别提高到160元和130元。全面实施城乡医疗救助，共救助1.8万人次、171.6万元，被列为全国城乡救助体系建设示范市。对种粮农民直接补贴等工作全面完成。解决了36个行政村4.5万人的饮水安全问题。改造中低产田5万亩，33个行政村4.82万人受益。完成43个扶贫开发项目，5012人实现脱贫。“两免一补”惠及困难家庭学生7.8万人次，确保了义务教育阶段的学生不因家庭困难而辍学。城乡教育发展进一步均衡协调，教育教学质量全面提升。文化事业和文化产业发展加快。社区文化、文化下乡、广场文化等活动丰富多彩。刘庄遗址成功入选“全国十大考古新发现”。两县新农合参合率均超过92%。接待游客485.6万人次，旅游直接收入8178万元。参加第三届全国体育大会地掷球和航模比赛取得2金1铜的好成绩，参加省十运会实现了金牌排名大幅前移。人口自然增长率控制在4.5‰以下，继续稳定了低生育水平。民主法制建设和社会稳定工作扎实推进。自觉接受人大及其常委会的监督，支持政协参政议政，坚持向人大报告工作、向政协通报情况，办理人大代表建议和政协提案293件。积极推进依法治市，有针对性地开展“一抓四进”法制宣传教育活动，取得新成效，荣获省法制宣传教育和依法治理工作先进市。社会主义荣辱观教育和精神文明创建活动继续深入。双拥共建活动积极开展，军政军民团结不断加强。扎实推进廉政建设，认真开展商业贿赂专项治理，反腐倡廉工作取得新成效。深入开展平安鹤壁创建工作，各类安全事故起数、死亡人数分别下降27.9%和12.7%，除交通事故外，全市没有发生一起安全生产死亡事故，确保了在全省的先进位次；连续3年现行命案全破，是全省现行命案保持全破记录时间最长的省辖市；信访工作继续保持全省先进位次，社会大局保持稳定平安，荣获全省平安建设先进市。

　　经济发展环境进一步改善。积极推进干部队伍建设和政府效能建设，全市广大干部职工创新工作、合作共事、加快发展的内在活力明显增强。进一步提高了政府的执行力和公信力，从严查处破坏发展环境的典型案件。整合“12345”效能监察投诉中心、市长热线、“12319”城建热线、市政府网站留言板，成为加强政府与企业、与群众沟通的有效渠道，并以此为平台，建立了对群众意见进行分类整理、交办跟踪、落实反馈的制度规范并予以实施，取得了良好的社会效果。创新服务机制，优化办事程序，基本建设阶段代办督办取得较好成效。在全省省辖市投资环境评价中，我市政府部门管理效率各项指标均名列第一。

　　各位代表！2006年我市经济社会发展取得了令人鼓舞的好成绩：全市生产总值、规模以上工业增加值、全社会固定资产投资、财政一般预算收入总量实现标志性跨越，主要指标增速保持全省先进位次；社会事业全面发展，和谐社会建设迈出坚实步伐，经济社会发展、城乡发展更趋协调；不少工作成为全省乃至全国的特色和亮点，9个方面的工作被确定为国家级示范或试点，17个方面的工作被确定为省级示范或试点，显示了蓬勃生机和发展活力。

　　这些成绩是在我市发展起点升高、约束因素增多、区域竞争加剧的情况下取得的，的确来之不易，饱含着全市广大干部群众的心血和汗水，值得全市人民为之振奋、为之自豪。这些成绩的取得，是省委、省政府和市委正确领导的结果，是市人大、市政协大力支持的结果，是全市人民努力奋斗的结果。在此，我代表市人民政府，向辛勤工作在全市各行各业的广大工人、农民、知识分子、干部、驻鹤部队指战员、武警官兵、公安民警以及社会各界人士致以崇高的敬意！向关心、支持我市现代化建设的朋友们表示衷心的感谢！

　　我们也清醒地看到，我市经济社会发展还存在一些困难和问题，政府工作还有不少需要改进和加强的地方：经济结构还需要进一步优化；第三产业发展相对滞后，高科技企业较少，社会事业发展还相对薄弱；影响社会和谐的因素仍然不少，还有许多关系群众切身利益的问题急待我们去解决；一些政府工作人员的工作能力、服务水平与新形势新任务和广大群众的要求还存在差距，一些部门工作作风还需进一步改进。对此，我们一定高度重视，采取有效措施认真加以解决。

　　二、2007年工作总体要求

　　2007年是我市深入贯彻科学发展观、全面落实市七次党代会精神的关键一年，科学把握当前大势、积极谋划好我市的发展十分重要。国家对中部崛起、循环经济发展、社会主义新农村建设、和谐社会建设等支持力度进一步加大，为我市又好又快发展提供了良好机遇和有利条件。特别是市七次党代会提出“加快建设新鹤壁、中原崛起争先锋”的奋斗目标，进一步调动了全市人民干事创业的热情，为我市又好又快发展提供了强大动力。在新的一年，我们要坚定信心，振奋精神，加大改革开放和创新力度，推进各项重点工作取得更大进展，全面提升整体实力和区域竞争力。

　　今年政府工作的总体要求是：以邓小平理论和“三个代表”重要思想为指导，按照省委省政府的总体部署和市七次党代会、市委经济工作会的总体要求，坚持以科学发展观统领经济社会发展全局，以循环经济、城乡一体化、和谐社会建设统筹各项工作，继续强力推进招商引资和大项目建设，加快产业结构优化升级和经济增长方式转变，加快工业化、城镇化和农业现代化，努力实现经济社会又好又快发展，在加快建设新鹤壁、中原崛起争先锋的新征程中迈出坚实步伐。

　　经济社会发展的主要预期目标是：全市生产总值增长15%；规模以上工业增加值增长22%；财政一般预算收入增长16%；全社会固定资产投资增长25%，其中城镇固定资产投资增长30%；社会消费品零售总额增长14%；到位市外直接投资超过80亿元、力争90亿元；外贸出口增长15%；万元生产总值能耗降低5.5%；化学需氧量、二氧化硫排放量分别削减3.8%和4%；城镇居民人均可支配收入增长12%，农民人均纯收入增长9%；新增城镇就业3万人；人口自然增长率控制在5‰以下。

　　三、大力发展循环经济，

　　努力推进产业结构优化升级，

　　加快经济增长方式转变

　　大力发展循环经济，加快建设国家循环经济示范市。把抓循环经济、抓环境保护与促进产业发展、提高企业效益更好地统一起来，更加注重整体的谋划、特色的打造、优势的发挥，更加注重政策创新完善和市场力量运用，更加注重新技术、新产品、新产业的开发引进，进一步丰富循环经济内涵、拓展外延。加强督促与服务，扎实推进循环经济项目建设。秸秆发电项目5月试运行，电厂二期脱硫项目6月建成，同力水泥余热发电项目8月试运行，鹤煤集团矿井水综合利用项目9月完工，电厂一期脱硫年内试运行，煤层气发电项目年内全部建成。新开工建设年产60万立方米粉煤灰陶粒和40万立方米陶粒混凝土砌块等20个项目。积极谋划推进污水处理厂中水回用、废旧物资处理等一批新项目。切实抓好16个循环经济示范企业建设。支持鹤煤集团、维恩克公司制定整体规划，争取用两年时间建成全国一流的循环经济示范企业。加快推进4个循环经济示范区建设，重点建设区内补链项目，拉长产业链条，强化产业间衔接，金山、六矿初步形成示范区域并发挥效应。把建筑节能、城市节能摆上重要位置，下大力气推进城市循环体系建设，确保取得实质性进展。依靠政府和市场的合力，加强与国内研发机构的联系合作，积极筹建循环经济研发机构，使我市循环经济在更高的技术平台上加快发展。着力推进太行全利公司环保节能型电炉炼钢成套装备、正道公司玉米收获机等优势项目，帮助企业引进战略伙伴、融通资金，尽快实现产业化。

　　切实抓好集约节约发展，加快建设资源节约型社会。把节能降耗及节水节地，作为大力发展循环经济、转变经济增长方式的着力点，作为建设资源节约型社会的重要内容，力求取得更大实效。切实抓好企业节能降耗工作。对重点能耗企业实行节能降耗目标管理。鼓励督促鹤煤集团等40户能耗大户，加快相关技术创新和工艺改造。积极引进节能服务公司，开展合同能源管理试点。建立合理用能评估和审查制度，加强节能降耗考核。下大力气推进建筑节能。新建居住建筑全面执行节能65%的设计标准，新建公共建筑全面执行节能50%的设计标准。结合市容市貌整治，综合运用政府支持、政策导向、市场融资等手段，有计划地推进既有公共建筑、居住建筑、公用事业等节能改造。科学合理地推进浅层地热利用。加快太阳能热水器普及。把推进建筑节能与供热收费政策改革结合起来，加快推进供热体制改革和供热计量收费工作，进一步做好城市集中供热工作。大力推广农业生产节水灌溉。积极推进矿井水再利用和中水回用。认真贯彻、严格执行国家土地法规政策，加强项目用地核实，把有限的建设用地指标用于最急需最关键的项目。在工业集中区大力推广使用多层标准厂房，提高容积率和单位土地面积投资强度。继续推进土地综合整治工作，加大农村建设用地整理力度。

　　全面加强环境保护和生态建设，加快建设环境友好型社会。认真落实环境影响评价等制度，使环保工作走在全省前列。继续加强饮用水源地保护，饮用水源地水质达标率保持100%。继续做好卫河流域水污染综合整治。深化工业污染防治，强力推进山城区化工企业搬迁和其它化工企业整治，对3家污染严重企业实施强制性清洁生产审核，对鹤煤集团、万和发电等企业实施清洁生产审核。淇滨污水处理厂竣工，市垃圾无害化处理场、市医疗废弃物处置中心和浚县、淇县生活垃圾处理场建成投用。采取加强污水和垃圾处理费征收、严格在线监控等措施，保证污水、垃圾处理企业的正常运行。抓好农村生活垃圾和污水处理试点工作。加快编制太行山生态保护规划，推进浅山生态绿化建设，积极谋划推进城市周边和市区、城镇建成区适宜地段的生态建设，完成人工造林8万亩以上。加大淇河生态保护力度和沿岸绿化、景观建设，加快打造生态休闲长廊。

　　四、继续推进招商引资

　　和大项目建设，进一步增强

　　经济实力和发展后劲

　　进一步提高招商引资质量，加快发展开放型经济。围绕我市经济发展总体思路、重点产业发展指向、工业集中区建设，继续大力实施开放带动主战略。在保持招商引资基本规模的基础上，进一步发挥专业招商的作用，更加注重招商引资的实际效果。围绕重点产业发展，力争60万吨甲醇、绿色包装、20万吨肉食加工、陶瓷系列产品加工、金属镁深加工等项目招商引资取得实质进展，确保引进项目规模和质量有更大提高。各县区、市直主要招商引资部门年内力争各引进1个以上投资超5亿元的项目，全市力争引进2个以上投资超10亿元的项目。进一步完善招商引资综合考评办法。加大对在谈重点项目的跟踪落实，加强对落地项目投资建设情况的督促检查，努力帮助建成企业解决生产经营中的困难和问题。更好地发挥企业的招商引资主体作用，提高以商招商水平。积极参加第二届中国中部投资贸易博览会等招商活动，高水平筹办中国(鹤壁)绿色包装产业发展研讨会、中国汽车配件产业论坛，利用建市50周年庆祝活动开展经贸洽谈和招商引资，力争取得较大成效。

　　继续强力推进大项目建设，持续增强发展潜力和后劲。在保持投资快速增长的同时，进一步优化投资结构，着力推进与重点产业发展紧密相关的大项目建设。加快推进46个在建重点项目。热电联供项目2号机组3月试运行。电厂三期两台机组分别于9月、12月投产发电。采煤沉陷综合治理项目完成在建住宅小区建设，开工建设牟山一区南区。继续做好移民安置等工作，确保盘石头水库汛后下闸蓄水。新开工18个对全市发展有重要促进作用的大项目。重点抓好60万吨甲醇、鹤辉高速公路、20万吨肉食加工、1000万件服装、绿色包装、煤矿机械、丹尼斯大卖场、高尔夫球具等项目的前期工作，争取早日开工建设。努力服务好长鹤泰铁路、京郑铁路客运专线、京珠高速公路拓宽、南水北调中线工程等项目实施，符合我市发展需要，确保项目建设在我市顺利推进。

　　提高产业规模度和聚集度，做大做强优势特色产业。进一步强化项目支撑，重点推进总投资157亿元的44个工业项目建设，加快八个特色产业的发展步伐，壮大产业规模，增强产业竞争力。鼓励企业加快做强做大，力争更多的企业进入省百户重点企业和50户高成长性企业盘子。继续做好融资工作，协助天海集团等企业上市，实现我市上市公司零的突破。加快企业研发中心建设，增强自主创新能力。积极实施知名品牌发展战略，加强标准化工作，引导扶持1—3家优势企业进入国家、省知名品牌企业行列。进一步谋划产业布局，完善工业集中区基础设施和服务功能，加快企业聚集，形成产业特色、规模效益和竞争优势。畅通企业与政府有关部门的对话沟通渠道，为企业家搭建更好的培训、交流、合作平台，努力造就一批具有战略思维、创新精神、现代经营管理能力和社会责任意识的企业家。

　　大力发展服务业，积极培育新的经济增长点。加大服务业改革创新力度，进一步提高服务业的层次和水平，使之成为我市经济发展新的重要增长点。完成服务业体制改革和创新试点实施方案及商贸、现代物流规划。加快瑞丰物流中心、城市配送体系建设，积极推进四季青农产品批发市场等项目建设，培育粮食、蔬菜、汽车等有一定基础的特色物流。深入推进金融生态建设，支持国有商业银行改革发展、提高效益；深化农村信用社改革；推进城市信用社增资扩股，争取成功申报商业银行；多渠道扩大市担保中心资本金规模，进一步发挥在中小企业、县域经济发展融资中的作用。加快旅游景区建设、改革和发展步伐，推进浮丘山、古灵山创建国家4A级旅游景区工作，搞好旅游资源整体开发推介，推动旅游业实现较大发展。鼓励发展信息服务、商务中介等新兴服务业。大力改造传统服务业，提升餐饮、商业的规模和档次。科学务实推进房地产开发建设，进一步增强建筑业、房地产业拉动经济发展的能力。继续毫不动摇地鼓励、支持和引导非公有制经济快速健康发展，进一步发挥非公有制经济在全市经济发展中的作用。

　　五、努力推进城乡一体化,

　　加快新型城镇化和新农村建设步伐

　　继续突出产业支撑、城镇辐射带动、基础设施向农村延伸、公共服务向农村覆盖、工业向园区集中、人口向城镇集中以及相关体制机制创新等重点工作，全方位、深层次推进城乡一体化，加快推进新型城镇化和新农村建设步伐。城镇化率达到46%。

　　加强新区、老区和两县县城建设与管理，加快新型城镇化步伐。坚持以新区为中心，老区、两县县城为支撑点，加快经济发展和城区扩张，进一步提高城市综合承载和辐射带动能力。新区紧紧围绕创建宜居城市的目标，进一步加强产业支撑，提高建设品位和管理水平，加快人口聚集。抓好精品示范项目，打造精品建筑、精品小区。建成淇河橡胶坝，高质量、高品位建设淇河公园和淇河新区段。完成二支渠西段改造，开工东段改造工程，逐步完善新区城市水系。积极推进体育中心、妇幼保健院等项目建设，建成湘江小学。新区南部两个村民安置小区一期工程竣工，大赉店村黄河路段改造全面完成，西郭庄、半坡店、大梁庄等旧村改造全面启动。老区着力加快产业结构优化升级，改善基础设施，下大力气抓好环境治理，切实解决道路遗撒和扬尘等问题，努力改善群众的生活质量和居住环境。两县加快提高县城的建设管理档次，增强人口吸纳力，在产业发展、县城建设、人口聚集等方面努力争取更大进展。乡镇特别是重点镇继续加快建设发展步伐。认真做好城市总体规划修编，提高新区、老区和两县县城规划的管理水平。全部完成乡镇总体规划修编、行政村规划编制任务。进一步创新理念、完善思路，提高城市管理水平，推行精细化管理。加强城市管理综合执法，提高城市管理队伍素质，促进城市管理水平再上新台阶。创新完善管理办法，进一步提高物业管理水平。积极推进村改居工作。

　　全面做好“三农”工作，加快推进新农村建设。大力发展现代农业，加快农村基础设施建设和社会事业发展。加快农业产业化进程和特色产业发展。大力扶持食品加工、养殖、种植龙头企业发展，加快农业规模化生产基地建设。引导、督促乡镇、村大力发展食品加工业和农业产业化配套产业。畜牧业产值、加工业产值分别增加10亿元和15亿元，畜牧业占农业总产值的比重达到63%。新增市级以上龙头企业12家。农民组织化程度提高到54%。认真落实各项支农惠农政策。加快农村基础设施建设。实施交通便民、饮水安全、沼气入户、生态环保、信息服务工程。建设农村道路130公里，进一步改善66个行政村道路状况；新建、改建大中型桥梁10座；25个乡镇客运站全部投入使用，全市行政村通班车覆盖率达到99%。推广单村集中供水、联村集中供水等模式，再解决5万人的饮水安全问题，农村自来水入户率达到68%。改造中低产田4万亩。解决4000人脱贫问题。新发展沼气1.5万户，农村沼气用户达到适宜建沼农户的35%以上。积极发展集中供应沼气。加快生态绿化、环境整治和面源污染治理。消除农村电网改造盲点村20个，新增通电户数600户，农村电话入户率达到68%，乡乡建有农业信息服务站。加快发展农村社会事业。建立覆盖全市农村的新型农村合作医疗制度，新建200所标准化村卫生室。新建6所乡镇文化站、46个示范性村文化大院，实施文化信息资源共享工程。建成20个农民体育健身工程。实现20户以下自然村村村通广播电视，广播电视人口综合覆盖率达到100%。深入推进农村综合改革，实施农村公益事业“一事一议”筹资筹劳办法。加快浚县农场改革发展步伐。建成20个乡镇级劳动力市场，建立覆盖全市的城乡统筹就业服务体系。完成2万名农村劳动力引导性培训、1.5万名技能性培训，保持20万名以上农村富余劳动力转移就业规模。

　　加快体制机制和工作创新，积极探索城乡一体化的新路子。注重把新农村建设与加快城镇化更好地结合起来，注重有关政策创新和完善，注重制度建设和强化管理，注重新模式的探索实践，进一步优化城乡协调发展的思路、政策和办法，通过创新为加快城乡一体化提供充足的动力和活力。积极探索建立村容村貌整治、基础设施建设、公共服务发展等方面的长效机制，探索推动土地依法流转、实现土地规模经营的新路子，探索鼓励农民到城镇居住就业、失地农民和农民工参加社会保险的政策和途径，探索制订城乡一体化工作评价体系等，努力为全省城乡一体化工作探索经验、作出示范。

　　六、高度关注和切实解决

　　关系群众切身利益的问题，

　　努力构建和谐社会

　　切实做好就业再就业工作。做好统筹城乡就业试点工作。加强公共就业服务体系建设，探索建立面向全体劳动者的培训、就业新机制。进一步整合培训资源，广泛开展职业培训、职业介绍和就业指导。切实做好下岗失业人员再就业工作，实现下岗失业人员再就业8100人。积极帮助“零就业”家庭、就业困难人员就业。注重解决高校毕业生和退伍转业军人就业问题。认真落实小额担保贷款、税费减免等就业再就业扶持政策。

　　进一步完善社会保障体系。推动保障由重点向全面延伸，努力构建覆盖城乡、统筹协调的社会保障体系。做好社会保险扩面和社会保险费征缴工作，新增城镇基本养老保险参保人数5500人、基本医疗保险参保人数5000人，确保养老保险金和失业保险金按时足额发放。积极推进原国有、集体破产和特困企业退休人员参加基本医疗保险工作。继续扩大社会救助覆盖面，加大社会救助强度。完善城乡低保制度，适当提高农村低保补差标准，确保城乡困难群众基本生活。完成10所敬老院建设，农村五保户集中供养率提高到35%。建成老年康复中心、儿童福利院。开工建设老年公寓、社会救助管理站。完善城乡医疗救助体系，健全对少年儿童、农民工大病医疗救助制度，加大对低保边缘群体、低收入群体的临时救助。丰富慈善工作内容和载体，创新捐赠方式，引导企业、群众积极参与经常性捐赠，推动慈善事业发展。认真组织实施公务员工资分配制度和规范公务员津贴补贴改革。稳步推进事业单位收入分配制度改革。增加离退休人员基本养老金。

　　扎实推进平安鹤壁建设。健全群众诉求表达、利益协调和权益保障机制，维护社会公平正义。完善公共应急管理体系建设，积极预防和妥善处置各类突发事件，努力提高公共安全保障能力。全面落实安全生产责任制，以地方煤矿为重点切实抓好重点领域和行业专项整治，积极做好农村和城市社区安全工作，推动安全形势继续好转。大力整顿和规范市场经济秩序，切实加强食品药品安全监管。认真落实信访工作责任制，健全人民调解、行政调解、司法调解相结合的矛盾纠纷调处机制。及时了解、努力解决群众关心的热点难点问题，坚决纠正损害群众切身利益的不正之风。健全社会治安防控体系，强化社会治安综合治理，深入开展命案侦破、打黑除恶、“两抢一盗”等专项斗争，最大限度遏制各类违法犯罪活动发生，进一步增强人民群众的安全感，保障人民群众安居乐业。

　　全面发展社会事业。积极推进教育教学改革和管理创新，进一步搞好教师培训，切实提高农村学校、城镇薄弱学校办学条件和教育水平，推进城乡教育均衡发展，提升全市总体教育质量和水平。继续做好“两免一补”工作，全部免除农村义务教育阶段学生的学杂费，对困难家庭学生免费提供课本并补助寄宿生生活费。建成特殊教育学校。重视和加强农村中小学图书室的投入、运转和管理。基本完成教育资源整合优化任务。维修改造农村中小学校舍3万平方米。更新100个困难村学校的课桌凳。进一步提高农村中小学现代远程教育工程应用水平。完成农村中学实验室改造工程。加快城乡学前三年教育普及步伐。加快职业教育资源整合和开放办学步伐，职业技术学院以创建国家示范性高职院校为契机，进一步提高教育教学水平。加强民办学校的管理服务，提高民办学校办学水平。加大科技创新力度，加强科技人才队伍建设，组织实施“十一五”国家粮食丰产科技工程、科技富民强县工程试点工作，进一步提高科技支撑经济发展的能力。加强公共卫生和医疗服务体系建设，提高重大疾病防控和医疗救治能力，加强乡、村两级和城市社区卫生服务建设，进一步提高医疗服务水平。整合医疗卫生资源，抓好医疗机构特色专科建设，鼓励发展特色医疗机构。加强医德医风建设，规范医疗服务行为。完善药品集中招标采购制度，降低药品价格。健全城乡卫生监管网络，推行市区一体化卫生监督模式。积极建设面向社区、群众的体育设施和场所，广泛开展全民健身运动。继续稳定低生育水平，提高出生人口素质。进一步做好民族宗教等工作。

　　加快发展文化事业和文化产业。进一步组织丰富多彩的群众文化活动。拓展文化中心特别是图书馆、群艺馆服务内涵，加快延伸面向县区群众的服务网络。高水平建设山城区文体中心。繁荣发展新闻出版事业。积极推进广播电视数字化进程。科学谋划和找准突破口，加快文化项目引进，打造优势文化产业品牌，扩大特色文化产业规模。积极发展浚县古庙会等民俗文化、地掷球等体育文化；加快黄河古陶等传统民间工艺品的开发，培育文化骨干企业；加强文化市场监管，对经营性网吧实施透明规范、严格有效的监管。扎实推进文化事业单位、经营性文化单位和文化管理体制改革。

　　加强精神文明和民主法制建设。把社会主义核心价值体系融入精神文明建设全过程，加强思想道德、和谐文化建设，加强社会公德、职业道德、家庭美德教育，深入开展“道德规范进万家、诚实守信万人行”活动，在全社会树立知荣辱、讲正气、树新风、促和谐的新风尚。加强青少年思想道德建设。深入开展“扫黄打非”。继续开展文明城市、文明景区“双创”活动，广泛开展文明行业、文明单位和文明村户创建活动。扎实推进依法治市，在全市广泛深入开展法制宣传教育活动，提高全民法律意识，营造人人自觉学法守法用法的社会氛围。认真落实监督法，严格依照法定权限和程序行使权力、履行职责。自觉接受人大及其常委会的法律监督、工作监督和政协的民主监督，严格执行人大及其常委会的决议、决定，支持人大代表依法行使职权，广泛听取各民主党派、工商联和无党派人士的意见和建议，不断改进政府工作。认真办理人大代表建议和政协提案。关心支持驻鹤解放军和武警部队建设，抓好民兵预备役和人防战备建设，开工建设武警支队教导队和民兵综合训练基地。认真落实优抚安置政策，深入开展双拥共建活动，巩固和发展军政军民团结。充分发挥工会、共青团、妇联等人民团体以及协会、商会等社团组织的桥梁纽带作用，自觉接受全市人民的监督。加强法律援助工作，依法保障公民特别是妇女、未成年人和残疾人等弱势群体的合法权益。

　　七、加强干部队伍作风建设

　　努力建设为民、务实、廉洁的政府

　　大力倡导加快发展、争当先锋的风气。进一步倡导勤于学习的风气，推进学习型机关建设，不断拓展广大干部职工的视野，提高业务素养和工作能力。进一步倡导勇于创新的风气，不断创新工作思路、领导方式、工作方法，激发和调动广大干部职工的工作热情。进一步倡导务实为民的风气，多做一些深入实际的调查研究，多办一些为广大群众排忧解难的实事。进一步倡导肯干实干的风气，增强责任心、事业心，争取各项主要工作都达到全省先进水平。

　　努力增强加快发展、争当先锋的能力。增强科学发展的能力，努力将科学发展观真正贯穿到各项工作中。增强借势发展的能力，更好地抓住机遇、乘势而为，在认真贯彻落实国家方针政策中求得新的发展。增强谋划全局的能力，提升各项工作的整体水平和整体发展形象。增强抓好落实的能力，健全工作制度，严格执行问责制，强化效能监察，加强审计监督，保证市委、市政府的决策部署真正落到实处。

　　进一步提高加快发展、争当先锋的管理服务水平。加快职能转变，提高社会管理和公共服务水平。加强行政服务中心和有关部门对外服务窗口的管理，延伸便民服务网络。继续做好一线工作人员的规范管理，改进服务效率和质量，提高政务公开水平，更好地为基层、企业和公众服务。始终牢记“两个务必”，深入推进政府系统廉政建设和反腐败工作，认真抓好政府工作人员的教育、监督和廉洁自律，继续开展商业贿赂专项治理和政风行风建设，严肃查处各类违纪违法案件。加快建立统一、规范、公开、公正的招投标市场监管新制度，从源头上遏制招投标活动中的腐败现象。进一步提高公务员管理的规范化、科学化水平，努力建设一支高素质的公务员队伍。

　　各位代表！加快建设新鹤壁、中原崛起争先锋，我们重任在肩、责无旁贷。在新的一年里，让我们在省委、省政府和市委的正确领导下，全面落实科学发展观，同心同德，奋发图强，以又好又快发展的优异成绩迎接党的十七大胜利召开，迎接建市50周年，迈向鹤壁更加美好的明天！
